# Dietary vitamin B9 intake linked to drug-resistant epilepsy risk in western Chinese adults: exploring underlying metabolomic mechanisms

**DOI:** 10.3389/fnut.2025.1723304

**Published:** 2025-12-19

**Authors:** Ling Zhang, Ling Liu

**Affiliations:** 1 Department of Neurology, West China Hospital, Sichuan University, Chengdu, China; 2 Department of Neurology, Chengdu Eighth People’s Hospital (Geriatric Hospital of Chengdu Medical College), Chengdu, China

**Keywords:** B vitamins, drug-resistant epilepsy, epilepsy, metabolomics, vitamin B1, vitamin B12, vitamin B6, vitamin B9

## Abstract

**Introduction:**

Approximately 30% of epilepsy patients develop drug-resistant epilepsy (DRE), underscoring the need for adjunctive therapies. While dietary interventions like the ketogenic diet show promise, evidence for specific micronutrients, particularly regarding differences between DRE and non-drug-resistant epilepsy (NRE), remains scarce. This study investigated B-vitamin intake and plasma metabolomic profiles in NRE versus DRE patients.

**Methods:**

We established a cohort of 330 adults with epilepsy (165 NRE, 165 DRE) from western China. Dietary intake of vitamins B1, B6, B9, and B12 was assessed. Multivariate logistic regression and restricted cubic splines (RCS) analyzed associations between vitamin intake and DRE risk. Stratified analyses evaluated consistency across subgroups. Untargeted metabolomics was performed on a subset of patients to identify differential metabolites and pathways.

**Results:**

In the fully adjusted model, only dietary vitamin B9 (folate) intake was significantly and inversely associated with DRE risk (OR: 0.78 per 100 μg, 95% CI: 0.64–0.94; *p* = 0.01). The RCS indicated a linear dose-response relationship (non-linearity *p* = 0.412). This protective association remained stable across various subgroups without significant interactions. Metabolomic analysis revealed distinct profiles between NRE and DRE, identifying key differential metabolites including decreased linoleic acid and tetrahydrofolyl-glutamate, and increased cortisol. Pathway analysis implicated alterations in linoleic acid metabolism, cortisol synthesis and secretion, and folate metabolism.

**Conclusion:**

This study identifies lower dietary vitamin B9 intake as an independent risk factor for DRE in a Chinese adult cohort, with each 100 μg increase conferring a 22% reduction in risk. The metabolomic findings provide potential mechanistic insights, linking folate intake to dysregulated stress hormone, inflammatory, and one-carbon metabolism pathways in DRE. These results highlight the potential of nutritional assessment and intervention in the management of drug-resistant epilepsy.

## 1 Introduction

Epilepsy is one of the most common serious neurological disorders worldwide, affecting approximately 51 million patients (1), and causing significant health and socioeconomic burdens. Particularly alarming is the fact that about 30% of epilepsy patients will develop drug-resistant epilepsy (DRE) (2), characterized by no response to at least two reasonably used antiepileptic drugs (ASMs). Patients with DRE not only face higher rates of disability and mortality but also often suffer from depression, cognitive impairment, and a significant decline in quality of life. The limitations of the existing ASMs in terms of efficacy and potential side effects make it particularly urgent to explore safe and effective alternative treatment strategies.

In recent years, dietary and nutritional interventions have shown great potential in the management of epilepsy. For instance, the ketogenic diet (KD), a high-fat, low-carbohydrate special diet, has been proven to effectively reduce the seizure frequency of some patients by regulating energy metabolism and reducing neuroinflammation (3–5). The success of this intervention has prompted researchers to further consider the role of specific micronutrients. Among various micronutrients, B vitamins–including B1 (thiamine), B6 (pyridoxine), B9 (folate), and B12 (cobalamin)–have attracted attention due to their fundamental roles in neural function, neurotransmitter synthesis, and inflammation regulation (6–9). Additionally, certain B vitamins (especially folate) may help reduce the teratogenic risk of specific ASMs (10, 11), further highlighting their importance in the comprehensive management of epilepsy.

Despite the above reasonable theoretical basis, systematic and evidence-based dietary guidelines for epilepsy patients are still lacking, especially regarding the clinical evidence of specific nutrient interventions such as B vitamins. A crucial knowledge gap is that there are almost no studies focusing on whether there are significant differences in the dietary intake of key B vitamins (B1, B6, B9, B12) between DRE patients and non-drug-resistant epilepsy (NRE) patients. This difference may provide a new perspective for understanding the pathological mechanism of DRE. To initially explore the biological basis behind this difference, metabolomics, as a technology capable of systematically analyzing small molecule metabolites, may provide clues for revealing the metabolic characteristics related to DRE. By initially presenting the different plasma metabolic profiles of DRE and NRE patients, we hope to discover potential metabolic pathway changes related to disease resistance.

Therefore, this study aims to achieve two goals through an adult epilepsy cohort in western China: Firstly, to systematically compare the differences in the dietary intake of key B vitamins (B1, B6, B9, B12) between NRE and DRE patients; Secondly, to conduct exploratory analysis using non-targeted metabolomics to initially identify potential differences in metabolites and related pathways between the two groups of patients, thereby providing preliminary evidence and new research directions for understanding the nutritional and metabolic mechanisms of DRE.

## 2 Materials and methods

### 2.1 Ethical requirements

This study has been formally approved by the Medical Ethics Committee of West China Hospital of Sichuan University. The entire research process strictly adheres to the ethical standards outlined in the Declaration of Helsinki. We ensure that all patients participating in this study or their family members have fully understood the research content and have voluntarily signed the informed consent form after being clearly informed of the potential risks and benefits. We are committed to strictly protecting the privacy and personal information of the participants and ensuring the objectivity and authenticity of the research results.

### 2.2 Data source

The self-established cohort selected 165 patients each with non-drug-resistant epilepsy (NRE) and drug-resistant epilepsy (DRE), aged 20–60 years, who visited the outpatient clinic of West China Hospital from September 2023 to January 2025, totaling 330 patients who participated in the study. Inclusion criteria for the self-established cohort: (1) Age 20–60 years; (2) Diagnosed with non-drug-resistant epilepsy, drug-resistant epilepsy, or symptomatic epilepsy by a neurologist, meeting the International League Against Epilepsy (ILAE) 2017 diagnostic criteria and classification for epilepsy. Exclusion criteria included: (1) Comorbid severe psychiatric diseases, malignant tumors, active infectious diseases; (2) Presence of severe comorbidities such as heart, liver, or kidney diseases; (3) Inability to cooperate with dietary data collection; (4) Pregnancy. From the self-established cohort, 6 NRE patients and 6 DRE patients were randomly selected for metabolomic testing. Randomization method: The 165 patients in the NRE subgroup were numbered 1–165, a random number sequence was generated using R language (version 4.3.3) and arranged in ascending order, the first 6 numbers were selected for inclusion; the same operation was repeated for the 165 patients in the DRE subgroup. The sampling process was completed by an independent statistician, and clinical researchers did not participate in number assignment.

### 2.3 Concept definitions

Drug-resistant epilepsy (DRE): According to seizure type, after rationally selecting and correctly using at least two well-tolerated anti-seizure medications as monotherapy or in combination, the duration of seizure freedom for the patient does not reach three times the longest pre-intervention inter-seizure interval or 1 year (whichever is longer) (12).

Symptomatic epilepsy: Epilepsy caused by a known etiology or brain injury. Usually accompanied by structural or metabolic causes, such as brain trauma, brain tumors, cerebrovascular disease, infection (e.g., encephalitis), congenital brain developmental abnormalities, etc (12).

### 2.4 Clinical data collection

Based on the established inclusion and exclusion criteria, eligible epilepsy patients were screened from the self-established cohort, and clinical data were collected using unified methods. To ensure data accuracy and completeness, we meticulously refined relevant information through face-to-face interviews or telephone inquiries with the patients themselves and family members who had long-term accompaniment.

The self-established cohort collected information including: (1) Demographic indicators: age, gender, education level, marital status, household income; (2) Clinical data: disease duration, age at onset, history of febrile seizures, family history, anti-seizure medication use, seizure frequency, Quality of Life in Epilepsy-31 inventory (QOLIE-31) (13), Neurological Disorders Depression Inventory for Epilepsy (NDDIE) (14), Mini-Mental State Examination (MMSE); (3) Comorbidities: hypertension, diabetes, stroke, coronary heart disease, chronic arthritis; (4) Nutritional indicators: The 24-h dietary recall method was used to obtain the daily intake of various foods for the study subjects. Based on the “China Food Composition Table,” the per capita intake of energy and various nutrients from various foods was calculated for the study subjects. We compared the calculated nutrient intake with the recommended intake (RNI) for adults as specified in the “Dietary Reference Intakes for Chinese Residents” to provide a clinical background reference.

To ensure the validity and reliability of the above questionnaires, we required all participants to complete the questionnaires in the order mentioned above. The scoring process was completed face-to-face by two trained specialists with the participants to ensure scoring accuracy and objectivity. Before scoring began, the physicians clearly explained the purpose of the scoring to the participants and obtained their consent. During the survey, we provided timely and clear answers to any questions raised by the participants to ensure they could accurately understand and answer the questions. For participants with lower education levels who could not complete the scale filling independently, we used a question-and-answer method for guidance.

### 2.5 Collection of metabolomic specimens

(1) Blood collection: 5 mL of fasting venous blood was collected, centrifuged to separate serum, aliquoted, and stored at −80 °C until testing. (2) Instruments and reagents: Untargeted metabolomic analysis was performed using liquid chromatography-mass spectrometry (LC-MS) (Majorbio MJX-8000). (3) Sample pretreatment: Serum samples were precipitated with acetonitrile, centrifuged, the supernatant was taken, freeze-dried, and reconstituted in a methanol-water (1:1) mixture. (4) Mass spectrometry parameters: Positive ion mode (ESI+) and negative ion mode (ESI−) were alternated for scanning, scan range m/z 50–1,000, resolution 70,000.

### 2.6 Covariate assessment

Covariates for the self-established cohort were based on previous literature and included gender, age, age at onset, marital status, average household income, education level, family genetic history, history of febrile seizures, disease duration, chronic comorbidities, smoking history, alcohol consumption history, physical activity, BMI, whether symptomatic epilepsy, comorbid depression, comorbid cognitive decline, quality of life, etc., were described. Finally, based on previous literature and baseline statistical differences, age at onset, average household income, education level, disease duration, physical activity, comorbid depression, comorbid cognitive decline, and quality of life were selected as covariates for inclusion in the regression model. Age at onset was divided into three groups: < 7 years, 7– < 18 years, ≥ 18 years; Marital status was divided into two groups: living alone (unmarried, divorced, widowed) or married; Average household income was divided into two groups: ≤ 5,000 yuan, > 5,000 yuan; Education level was divided into three groups: < 9 years, 9– < 12 years, ≥ 12 years; Disease duration was divided into three groups: < 5 years, 5– < 10 years, ≥ 10 years; Physical activity was divided into moderate-low activity intensity (basically inactive or exercise once per week) and moderate-high activity intensity (exercise more than twice per week); Comorbid depression was defined as a score > 12 on the NDDIE scale; Comorbid cognitive decline was defined as a score < 26 on the MMSE scale; Quality of life was the score on the QOLIE-31; BMI was divided into three groups using 18 and 25 as boundaries; Chronic comorbidities included any of hypertension, diabetes, coronary heart disease, stroke, or chronic arthritis.

### 2.7 Statistical methods

This study used R open-source software version 4.3.3 and Free Statistics software version 1.9 for comprehensive statistical analysis. A descriptive study was conducted for all participants, and a two-tailed test *P*-value less than 0.05 was considered statistically significant. The Kolmogorov–Smirnov test was used to determine if continuous variables were normally distributed. Due to scale issues, when vitamin B9 in the self-established cohort was analyzed as a continuous variable, its value was divided by 100, with units of 100 μg/unit. Normally distributed variables are expressed as (mean ± standard deviation), and skewed variables are expressed as median (interquartile range) [M (P25, P75)]. Categorical variables are expressed as percentages. One-way ANOVA was used for normally distributed variables, the Kruskal–Wallis test for skewed distributions, the chi-square test for categorical variables, and the *t*-test for continuous variables. Multivariate logistic regression was used to determine the correlation between various factors and the outcome, and multiple models were used to adjust for confounding factors in the multivariate regression. Logistic subgroup analysis was used to judge interactions between subgroups and assess the stability of this correlation across different subgroups. Restricted cubic spline (RCS) regression was used for linearity testing to explore whether quantitative relationships exist between each nutritional factor and the occurrence of drug-resistant epilepsy. During metabolomic detection, the R package ropls was used for missing value imputation (median), normalization (Probabilistic Quotient Normalization, PQN), and QC sample RSD filtering (RSD < 30%). Multivariate statistical methods (PLS-DA) combined with univariate analysis (*t*-test, *P* < 0.05, VIP > 1.0) were used to screen differential metabolites between groups. The KEGG and HMDB databases were used to annotate differential metabolites, and pathway enrichment analysis was performed using MetaboAnalyst 5.0 (hypergeometric test, *P* < 0.05).

## 3 Results

### 3.1 General clinical characteristics

The self-established cohort study included 330 epilepsy patients, comprising 165 NRE cases and 165 DRE cases. The mean age ± SD of participants was 32.6 ± 10.8 years, 158 (47.9%) were female, and 46.4% had an education level exceeding 12 years. At baseline, DRE patients were more likely to have the following characteristics: lower education level, lower household income, less physical activity, younger age at onset, longer disease duration, higher proportions of comorbid depression and cognitive impairment, lower QOLIE-31 quality of life scores, and lower dietary intake of vitamin B1 and B9. The Recommended Nutrient Intake (RNI) for vitamin B1 in Chinese adults is 1.4 mg per day for men and 1.2 mg per day for women; the RNI for vitamin B9 is 400 μg DFE per day. In this cohort, the average intake of the DRE group was significantly lower than the RNI, while the NRE group was closer to the RNI (see Table 1).

**TABLE 1:** Baseline characteristics by DRE classification.

Characteristics	Total (*n* = 330)	NRE (*n* = 165)	DRE (*n* = 165)	*P*
**Gender, *n* (%)**				1
Male	172 (52.1)	86 (52.1)	86 (52.1)	
Female	158 (47.9)	79 (47.9)	79 (47.9)	
**Age, years, mean ± SD**	32.6 ± 10.8	32.9 ± 12.0	32.2 ± 9.5	0.526
**Education, years, *n* (%)**				0.015
< 9	118 (35.8)	48 (29.1)	70 (42.4)	
9–< 12	59 (17.9)	28 (17)	31 (18.8)	
≥ 12	153 (46.4)	89 (53.9)	64 (38.8)	
**Marital status, *n* (%)**				0.27
Single	176 (53.3)	83 (50.3)	93 (56.4)	
Married	154 (46.7)	82 (49.7)	72 (43.6)	
**Household income (RMB), *n* (%)**				< 0.001
≤ 5,000	193 (58.5)	67 (40.6)	126 (76.4)	
> 5,000	137 (41.5)	98 (59.4)	39 (23.6)	
**Smoking status, *n* (%)**				0.76
Never	292 (88.5)	146 (88.5)	146 (88.5)	
Current	30 (9.1)	16 (9.7)	14 (8.5)	
Former	8 (2.4)	3 (1.8)	5 (3)	
**Alcohol intake, *n* (%)**				0.166
No	310 (93.9)	152 (92.1)	158 (95.8)	
Yes	20 (6.1)	13 (7.9)	7 (4.2)	
**Body mass index, kg/m^2^, *n* (%)**				0.573
< 18	22 (6.7)	9 (5.5)	13 (7.9)	
18– < 25	210 (63.6)	104 (63)	106 (64.2)	
≥ 25	98 (29.7)	52 (31.5)	46 (27.9)	
**Physical activity, *n* (%)**				< 0.001
Low-to-moderate	147 (44.5)	55 (33.3)	92 (55.8)	
High	183 (55.5)	110 (66.7)	73 (44.2)	
**Chronic comorbidities, *n* (%)**				0.48
No	294 (89.1)	145 (87.9)	149 (90.3)	
Yes	36 (10.9)	20 (12.1)	16 (9.7)	
**History of febrile seizures, *n* (%)**				0.09
No	306 (92.7)	157 (95.2)	149 (90.3)	
Yes	24 (7.3)	8 (4.8)	16 (9.7)	
**Family history of epilepsy, *n* (%)**				1
No	324 (98.2)	162 (98.2)	162 (98.2)	
Yes	6 (1.8)	3 (1.8)	3 (1.8)	
**Symptomatic epilepsy, *n* (%)**				0.557
No	274 (83.0)	139 (84.2)	135 (81.8)	
Yes	56 (17.0)	26 (15.8)	30 (18.2)	
**Age at onset, years, *n* (%)**				0.004
< 7	30 (9.1)	8 (4.8)	22 (13.3)	
7–<18	123 (37.3)	56 (33.9)	67 (40.6)	
≥ 18	177 (53.6)	101 (61.2)	76 (46.1)	
**Disease duration, *n* (%)**				< 0.001
< 5	94 (28.5)	70 (42.4)	24 (14.5)	
5– < 10	82 (24.8)	46 (27.9)	36 (21.8)	
≥ 10	154 (46.7)	49 (29.7)	105 (63.6)	
**Depression, *n* (%)**				< 0.001
No	234 (70.9)	143 (86.7)	91 (55.2)	
Yes	96 (29.1)	22 (13.3)	74 (44.8)	
**Cognitive impairment, *n* (%)**				< 0.001
No	225 (68.2)	138 (83.6)	87 (52.7)	
Yes	105 (31.8)	27 (16.4)	78 (47.3)	
**QOLIE-31 score, mean ± SD**	65.9 ± 10.8	70.5 ± 10.7	61.3 ± 8.9	< 0.001
Seizure worry	5.6 ± 1.1	5.9 ± 1.0	5.2 ± 1.1	< 0.001
Overall quality of life	8.1 ± 2.1	8.9 ± 2.1	7.3 ± 1.8	< 0.001
Emotional wellbeing	9.6 ± 2.0	10.4 ± 1.8	8.8 ± 1.9	< 0.001
Energy/fatigue	7.7 ± 1.4	8.3 ± 1.2	7.1 ± 1.4	< 0.001
Cognitive functioning	19.3 ± 2.9	20.4 ± 3.0	18.1 ± 2.4	< 0.001
Medication effects	2.0 ± 0.6	2.1 ± 0.5	2.0 ± 0.6	0.003
Social functioning	13.5 ± 2.9	14.4 ± 3.2	12.6 ± 2.3	< 0.001
Vitamin B1 intake (mg/d), mean ± SD	1.2 ± 0.4	1.2 ± 0.4	1.1 ± 0.3	0.002
Vitamin B6 intake (mg/d), mean ± SD	2.7 ± 0.8	2.8 ± 0.8	2.7 ± 0.8	0.518
Vitamin B9 (folate) intake (mcg/d), mean ± SD	341.6 ± 156.7	363.1 ± 152.9	320.2 ± 158.0	0.013
Vitamin B12 intake (mcg/d), mean ± SD	3.4 ± 3.6	3.7 ± 4.3	3.1 ± 2.7	0.168

Continuous variables are expressed as mean ± SD.

### 3.2 Multivariate logistic regression analysis of differences in B vitamins between NRE and DRE

**FIGURE 1:**
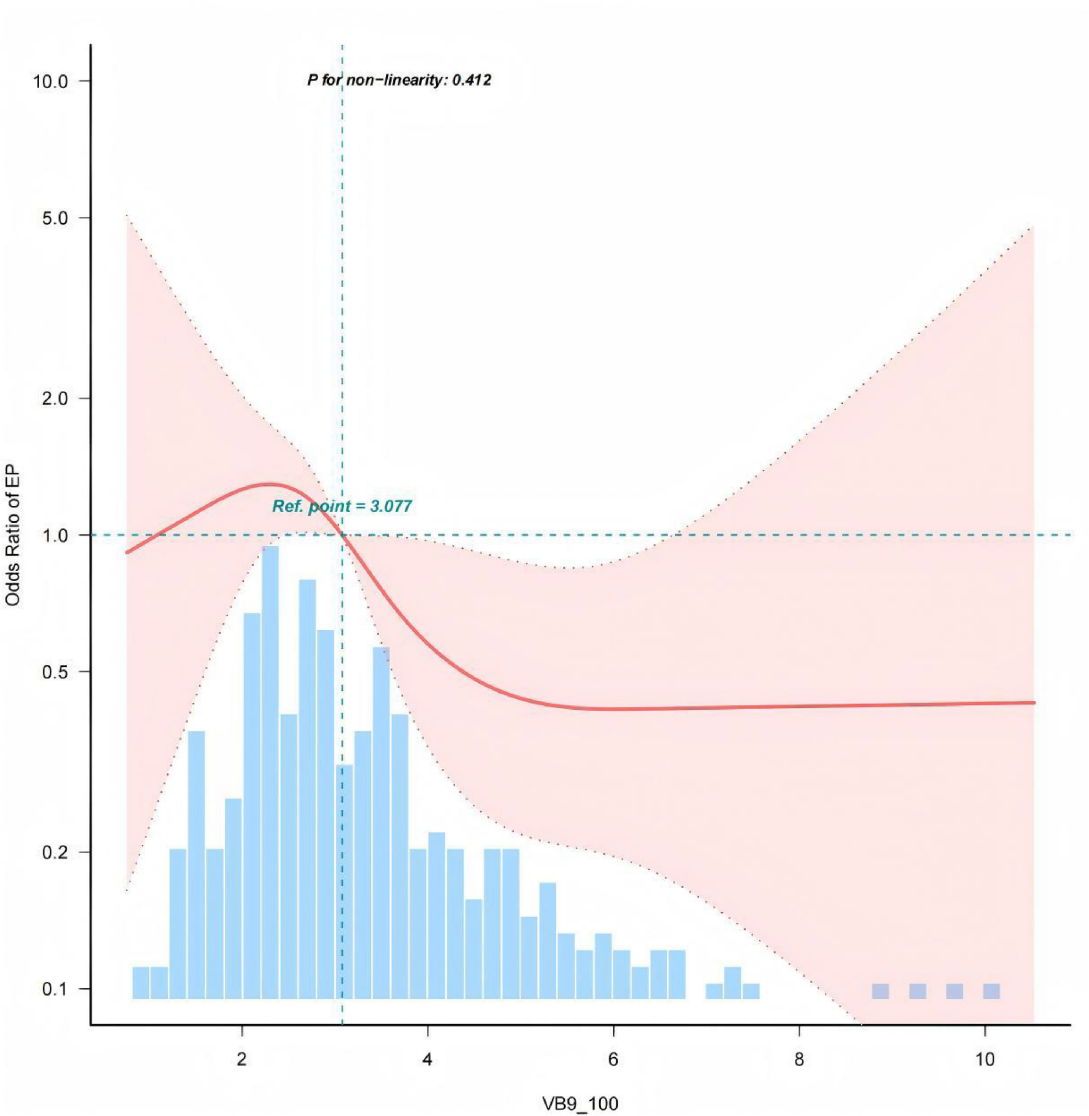
Association between vitamin B9 intake and the odds ratio of DRE. The solid and dashed lines represent the predicted values and the 95% confidence intervals, respectively. Adjusted for age at onset, years of education, household income, disease duration, physical activity, quality of life, comorbid depression, and comorbid cognitive impairment.

In the self-established epilepsy cohort, the results of the multivariate logistic regression analysis for the relationship between dietary intake of vitamins B1, B6, B9, B12 and the risk of drug-resistant epilepsy are shown in Table 2. In the unadjusted model, there were significant independent negative associations between vitamin B1 intake, vitamin B9 intake and the risk of drug-resistant epilepsy (OR: 0.36, 95% CI: 0.19–0.7; *p* = 0.002), (OR: 0.83, 95% CI: 0.72–0.96; *p* = 0.014), respectively; the differences were statistically significant; there were no significant independent associations between vitamin B6 intake, vitamin B12 intake and the risk of drug-resistant epilepsy (OR: 0.91, 95% CI: 0.69–1.21; *p* = 0.517), (OR: 0.96, 95% CI: 0.9–1.02; *p* = 0.177); In Model 1, after adjusting for demographic characteristics (age at onset, education level, household income), the significant independent negative associations between vitamin B1 intake, vitamin B9 intake and the risk of drug-resistant epilepsy remained (OR: 0.38, 95% CI: 0.19–0.78; *p* = 0.006), (OR: 0.84, 95% CI: 0.72–0.97; *p* = 0.022), and the differences remained statistically significant; In Model 2, after adjusting for demographic characteristics, disease duration, and physical activity, the significant independent negative associations between vitamin B1 intake, vitamin B9 intake and the risk of drug-resistant epilepsy remained (OR: 0.38, 95% CI: 0.18–0.79; *p* = 0.01), (OR: 0.83, 95% CI: 0.7–0.97; *p* = 0.023), and the differences remained statistically significant; Model 3 added comorbid depression, comorbid cognitive decline, and quality of life to Model 2 for comprehensive adjustment. The adjusted results showed that the negative association between vitamin B9 intake and the risk of drug-resistant epilepsy remained stable (OR: 0.79, 95% CI: 0.66–0.95; *p* = 0.012), while the negative association between vitamin B1 intake and the risk of drug-resistant epilepsy was not statistically significant (OR: 0.6, 95% CI: 0.26–1.38; *p* = 0.227). The RCS relationship between vitamin B9 and the risk of drug-resistant epilepsy is shown in Figure 1. When considering all confounding covariates, vitamin B9 intake was negatively associated with the risk of drug-resistant epilepsy (non-linearity: *p* = 0.412).

**TABLE 2:** Multivariable logistic regression analysis of B vitamins and DRE.

Variable	*n* = 330	Unadjusted	Model 1	Model 2	Model 3
Vitamin B1	OR (95% CI)	0.36 (0.19–0.7)	0.38 (0.19–0.78)	0.38 (0.18–0.79)	0.6 (0.26–1.38)
	*P*	0.002	0.006	0.01	0.227
Vitamin B6	OR (95% CI)	0.91 (0.69–1.21)	0.91 (0.67–1.23)	0.86 (0.62–1.19)	0.9 (0.63–1.3)
	*P*	0.517	0.543	0.366	0.585
Vitamin B9	OR (95% CI)	0.83 (0.72–0.96)	0.84 (0.72–0.97)	0.83 (0.7–0.97)	0.79 (0.66–0.95)
	*P*	0.014	0.022	0.023	0.012
Vitamin B12	OR (95% CI)	0.96 (0.9–1.02)	0.96 (0.89–1.03)	0.94 (0.87–1.01)	0.96 (0.88–1.05)
	*P*	0.177	0.274	0.095	0.379

Model 1: Adjusted for age at onset, years of education, and household income. Model 2: Adjusted for age at onset, years of education, household income, disease duration, and physical activity. Model 3: Adjusted for age at onset, years of education, household income, disease duration, physical activity, quality of life, comorbid depression, and comorbid cognitive impairment. BMI, body mass index; DII, Dietary Inflammatory Index; OR, odds ratio; CI, confidence interval.

### 3.3 Subgroup analysis

**FIGURE 2:**
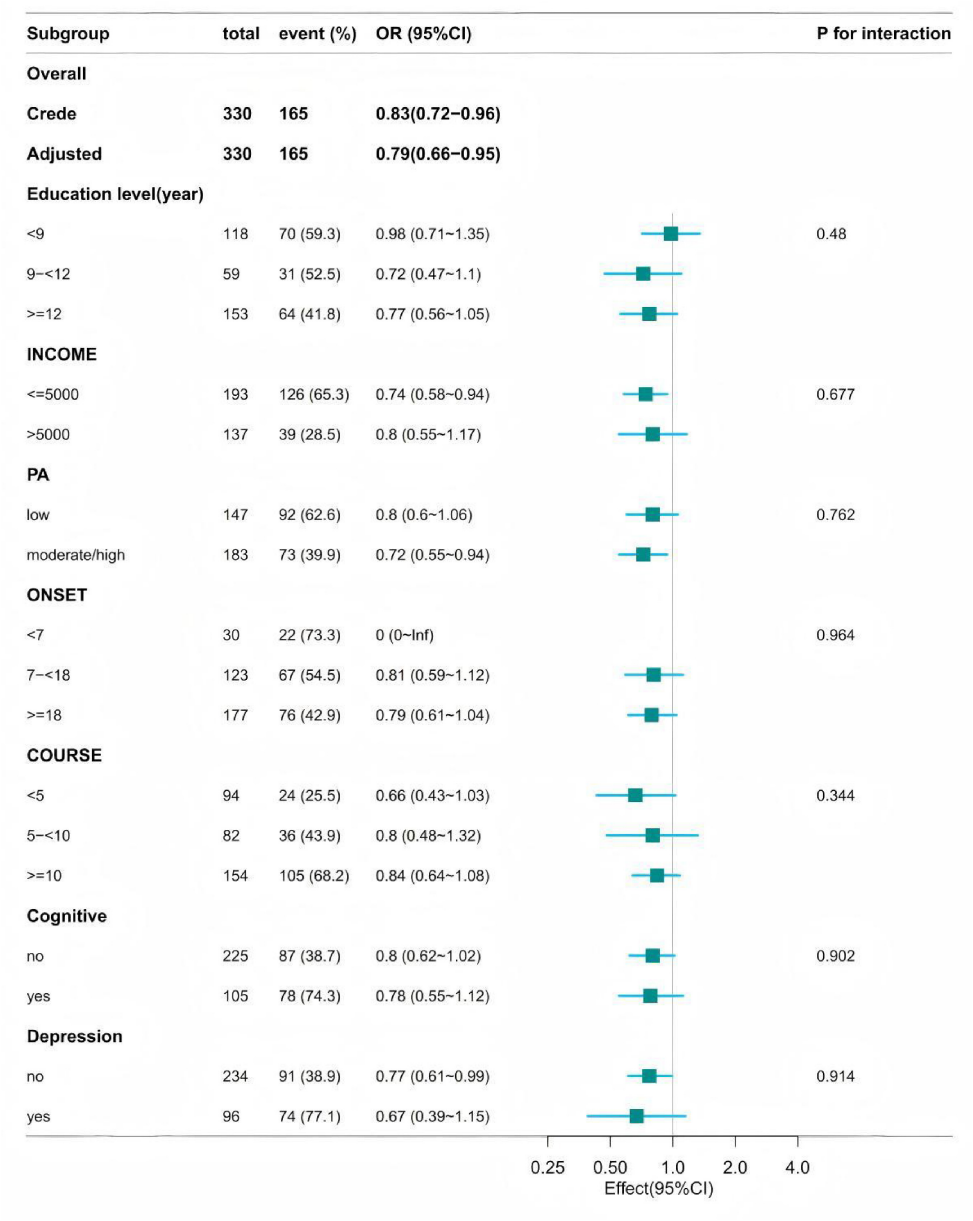
Subgroup analysis of the association between vitamin B9 intake and the risk of DRE. All strata were additionally adjusted for all other variables (age at onset, education level, household income, physical activity, disease duration, comorbid depression, comorbid cognitive impairment, and quality of life).

In the self-established epilepsy cohort, to determine whether the relationship between dietary vitamin B9 intake and the risk of drug-resistant epilepsy was consistent across subgroups, we performed stratified and interaction analyses. Age was dichotomized using 40 years as the cutoff and included in the subgroup analysis. As shown in Figure 2, stratification was performed based on covariates with a *P*-value < 0.05 in univariate analysis (education level, household income, physical activity, age at onset, disease duration, comorbid depression, comorbid cognitive impairment). In the groups with low household income (OR: 0.74, 95% CI: 0.58–0.94), higher physical activity (OR: 0.66, 95% CI: 0.49–0.89), higher age at onset (OR: 0.71, 95% CI: 0.51–0.98), shorter disease duration (OR: 0.51, 95% CI: 0.27–0.99), no comorbid cognitive impairment (OR: 0.75, 95% CI: 0.58–0.98), and no comorbid depression (OR: 0.77, 95% CI: 0.61–0.99), dietary vitamin B9 intake was significantly negatively associated with the risk of developing (drug-resistant) epilepsy. In the group with age at onset < 7 years, an OR value of 0.01 with a 95% CI of 0-lnf occurred, considered likely due to the small sample size in this group, preventing a reliable conclusion. When testing for interactions between subgroups using the likelihood ratio test, we found no statistically significant interactions in any subgroup.

### 3.4 Metabolomic analysis results

#### 3.4.1 General clinical characteristics

This metabolomics cohort included a total of 12 epilepsy patients, equally divided into DRE and NRE groups (*n* = 6 each) (see Table 3). The two groups showed no statistically significant differences (all *P* > 0.05) in basic demographic characteristics (e.g., gender, age, education, marital status, household income) or most clinical features (e.g., smoking and alcohol history, physical activity, chronic comorbidities, history of febrile seizures, family history of epilepsy, proportion of symptomatic epilepsy, age at onset, and disease duration), indicating well-balanced baseline characteristics. It is worth noting that patients in the DRE group appeared to have a higher prevalence of comorbid depression (DRE: 50% vs. NRE: 0%, *P* = 0.182) and a different BMI distribution, although these differences did not reach statistical significance, likely due to the small sample size.

**TABLE 3:** Baseline characteristics of the metabolomics cohort stratified by refractory epilepsy (DRE) status.

Characteristics	Total (*n* = 12)	NRE (*n* = 6)	DRE (*n* = 6)	*P*
**Gender, *n* (%)**				0.567
Male	6 (50.0)	4 (66.7)	2 (33.3)	
Female	6 (50.0)	2 (33.3)	4 (66.7)	
**Age, years, *n* (%)**				1
20– < 40	9 (75.0)	4 (66.7)	5 (83.3)	
40–60	3 (25.0)	2 (33.3)	1 (16.7)	
**Education, years, *n* (%)**				1
< 9	5 (41.7)	2 (33.3)	3 (50)	
9–< 12	2 (16.7)	1 (16.7)	1 (16.7)	
≥ 12	5 (41.7)	3 (50)	2 (33.3)	
**Marital status, *n* (%)**				1
Single	3 (25.0)	1 (16.7)	2 (33.3)	
Married	9 (75.0)	5 (83.3)	4 (66.7)	
**Household income (RMB), *n* (%)**				0.567
≤ 5,000	6 (50.0)	2 (33.3)	4 (66.7)	
> 5,000	6 (50.0)	4 (66.7)	2 (33.3)	
**Smoking status, *n* (%)**				0.455
Never	10 (83.3)	4 (66.7)	6 (100)	
Current	2 (16.7)	2 (33.3)	0 (0)	
Former				0.455
**Alcohol intake, *n* (%)**	10 (83.3)	4 (66.7)	6 (100)	0.178
No	2 (16.7)	2 (33.3)	0 (0)	
Yes				
**BMI, kg/m^2^, *n* (%)**	2 (16.7)	1 (16.7)	1 (16.7)	0.545
< 18	7 (58.3)	5 (83.3)	2 (33.3)	
18– < 25	3 (25.0)	0 (0)	3 (50)	
≥ 25				
**Physical activity, *n* (%)**	8 (66.7)	3 (50)	5 (83.3)	1
Low-to-moderate	4 (33.3)	3 (50)	1 (16.7)	
High				
**Chronic comorbidities, *n* (%)**	11 (91.7)	5 (83.3)	6 (100)	1
No	1 (8.3)	1 (16.7)	0 (0)	
Yes				
**History of febrile seizures, *n* (%)**	12 (100.0)	6 (100)	6 (100)	1
No				
Yes	12 (100.0)	6 (100)	6 (100)	
**Family history of epilepsy, *n* (%)**				1
No	8 (66.7)	4 (66.7)	4 (66.7)	
Yes	4 (33.3)	2 (33.3)	2 (33.3)	
**Symptomatic epilepsy, *n* (%)**				0.539
No	2 (16.7)	0 (0)	2 (33.3)	
Yes	5 (41.7)	3 (50)	2 (33.3)	
**Age at onset, years, *n* (%)**	5 (41.7)	3 (50)	2 (33.3)	1
< 7				
7–< 18	2 (16.7)	1 (16.7)	1 (16.7)	
≥ 18	1 (8.3)	1 (16.7)	0 (0)	
**Disease duration, *n* (%)**	9 (75.0)	4 (66.7)	5 (83.3)	0.567
< 5				
5–< 10	6 (50.0)	4 (66.7)	2 (33.3)	
≥ 10	6 (50.0)	2 (33.3)	4 (66.7)	
**Depression, *n* (%)**				0.182
No	9 (75.0)	6 (100)	3 (50)	
Yes	3 (25.0)	0 (0)	3 (50)	

#### 3.4.2 Differential metabolite analysis

**FIGURE 3:**
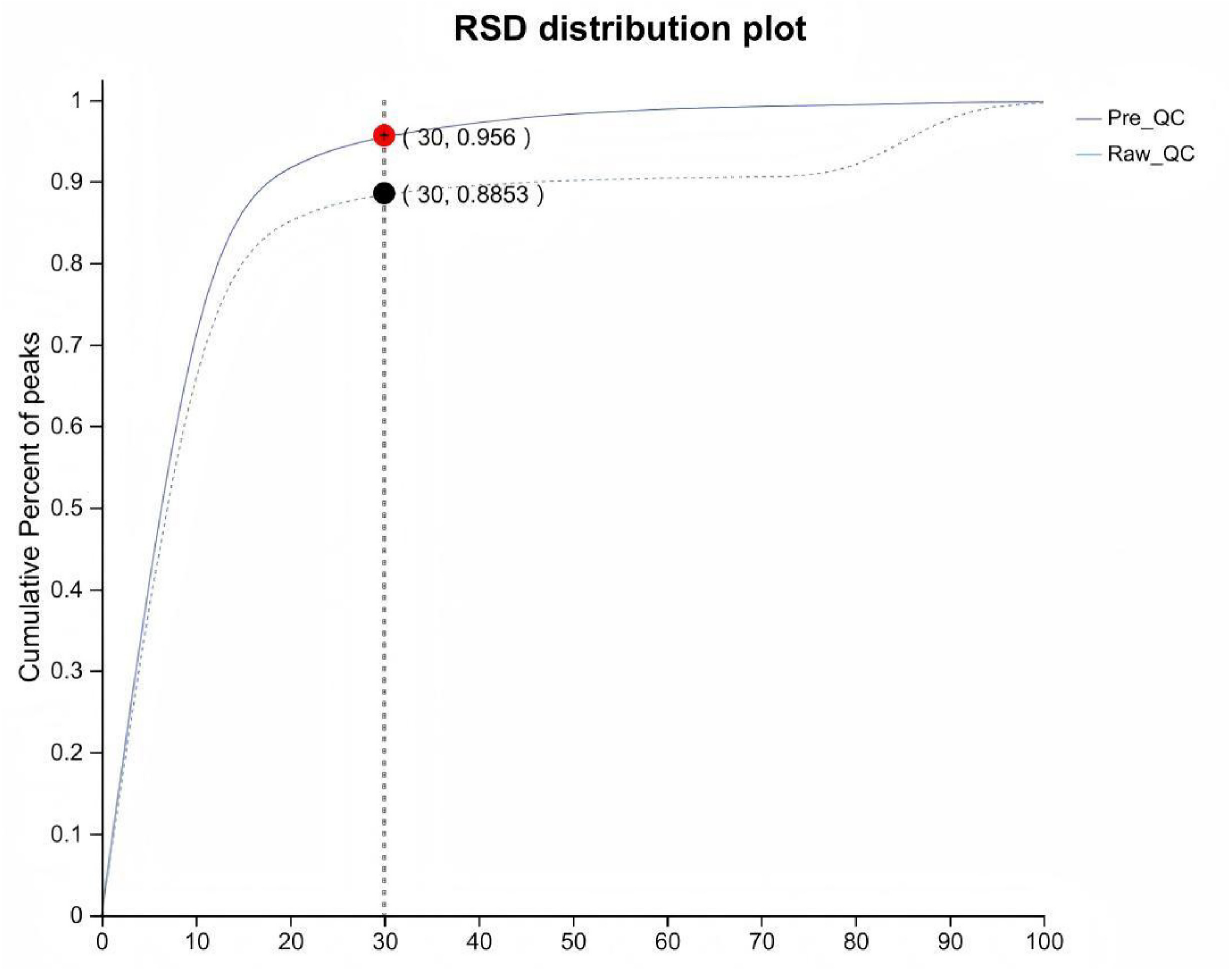
Quality control (QC) sample evaluation plot.

In the data preprocessing stage, the cumulative proportion of quality control (QC) samples with a relative standard deviation (RSD) exceeding 70% indicated reliable data quality, meeting the standards for metabolomic analysis. This step ensured the accuracy and reproducibility of subsequent analyses (see Figure 3).

**FIGURE 4:**
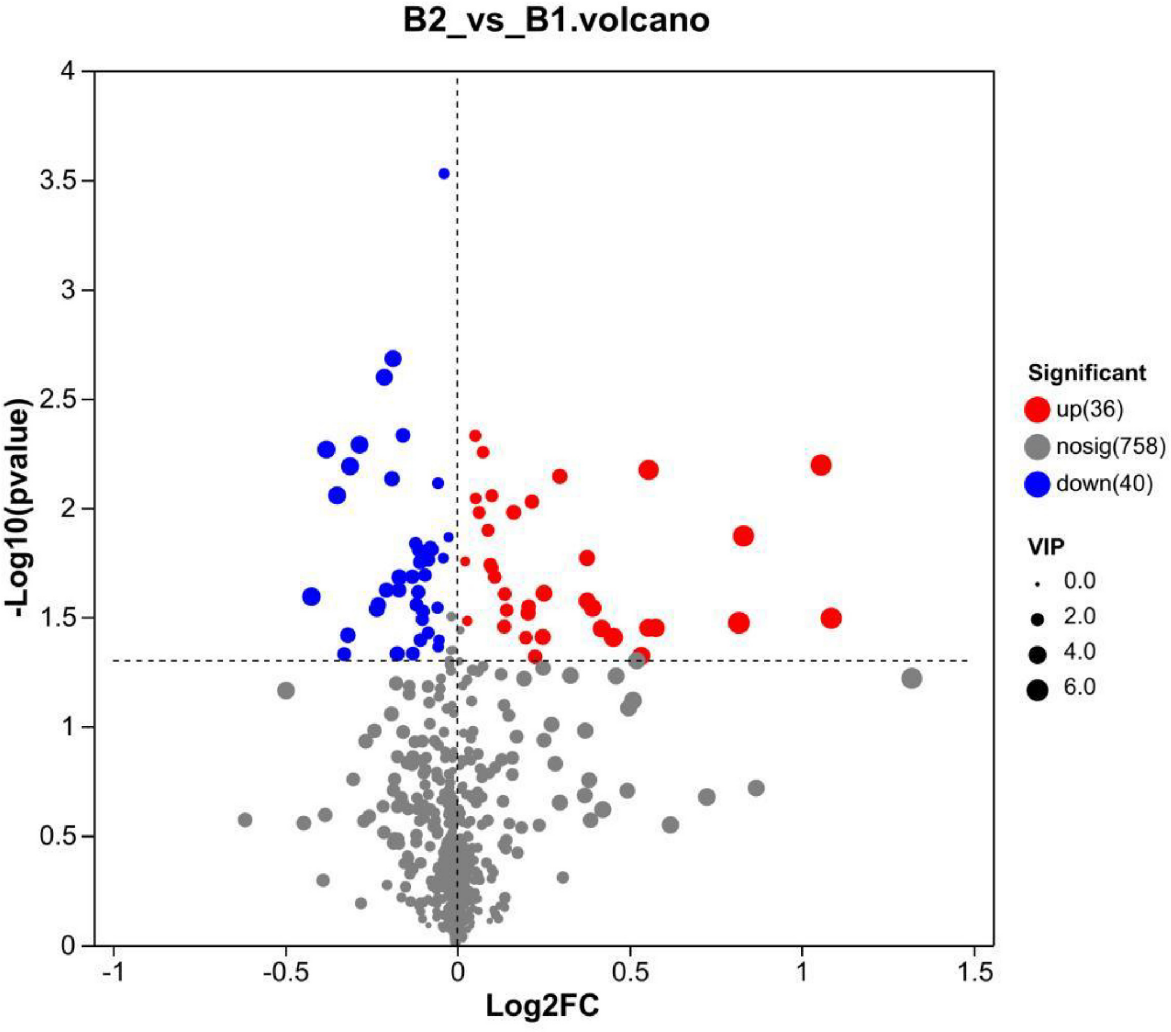
Volcano plot of differential metabolites. The *x*-axis represents the fold change of metabolite expression between the two groups (log2FC), while the *y*-axis represents the statistical significance of the differential expression [–log10(*P*-value)]. Higher values on the *y*-axis indicate more significant differential expression. B1, non-drug-resistant epilepsy (NRE); B2, drug-resistant epilepsy group (DRE).

**FIGURE 5:**
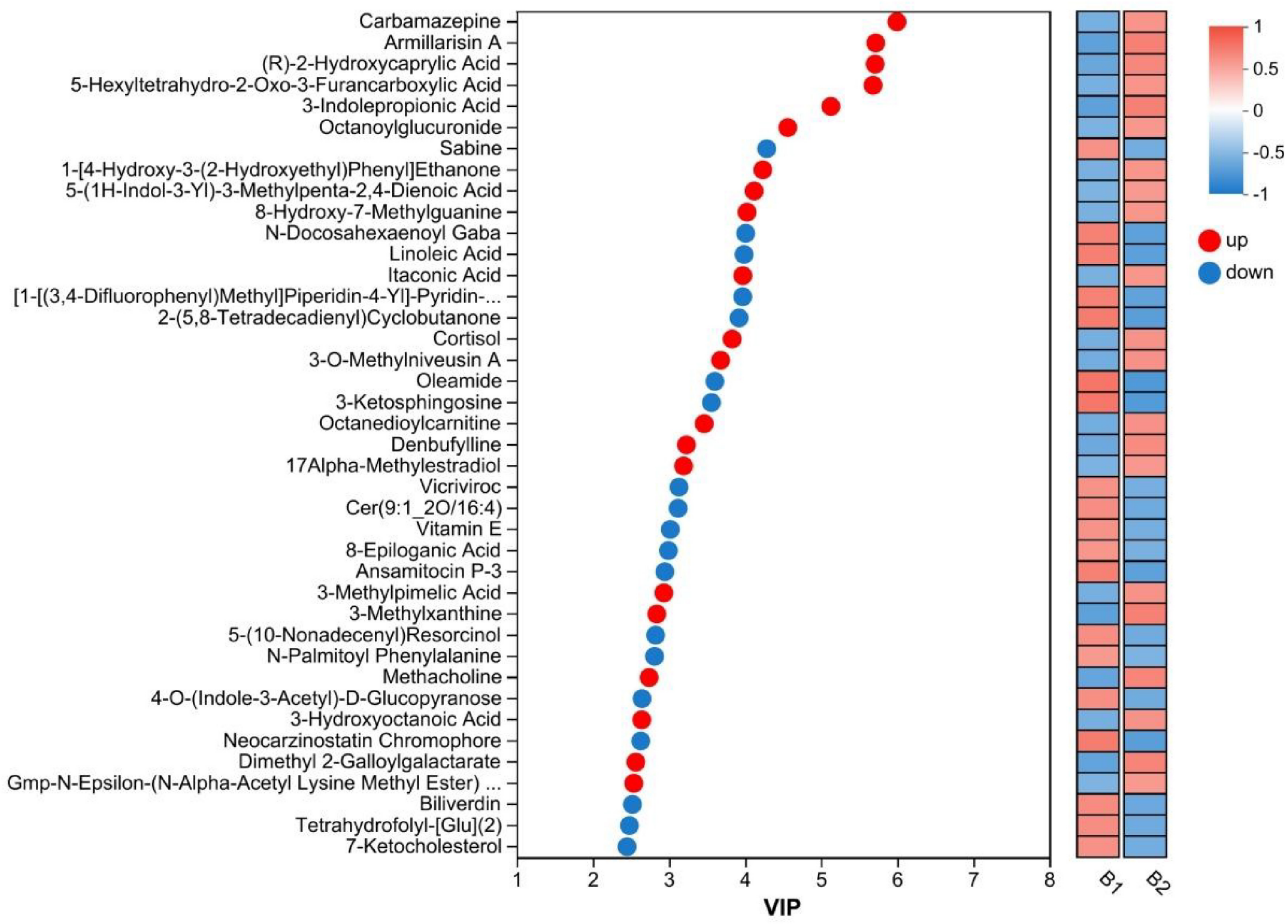
VIP plot of differential metabolites. VIP: variable importance in projection. B1, non-drug-resistant epilepsy (NRE); B2, drug-resistant epilepsy group (DRE).

Based on the VIP values from the PLS-DA model, we identified 76 significantly differential metabolites (VIP > 1.0, *P* < 0.05). These metabolites showed significant upregulation or downregulation trends between the non-drug-resistant epilepsy (NRE) and drug-resistant epilepsy (DRE) groups (see Figure 4). [Upregulated ones included: Carbamazepine (VIP value 5.997, *p* = 0.034), Armillarin A (VIP value 5.718, *p* = 0.006), (R)-2-Hydroxycaprylic acid (VIP value 5.711, *p* = 0.001), Cortisol (VIP value 3.83, *p* = 0.029), etc.; Downregulated ones included: Sabinene (VIP value 4.285, *p* = 0.026), N-Docosahexaenoyl GABA (VIP value 4.009, *p* = 0.006), Linoleic acid (VIP value 3.987, *p* = 0.005), Tetrahydrofolyl-glutamate (VIP value 2.48, *p* = 0.021), etc (see Figure 5).

#### 3.4.3 Metabolic pathway enrichment

**FIGURE 6:**
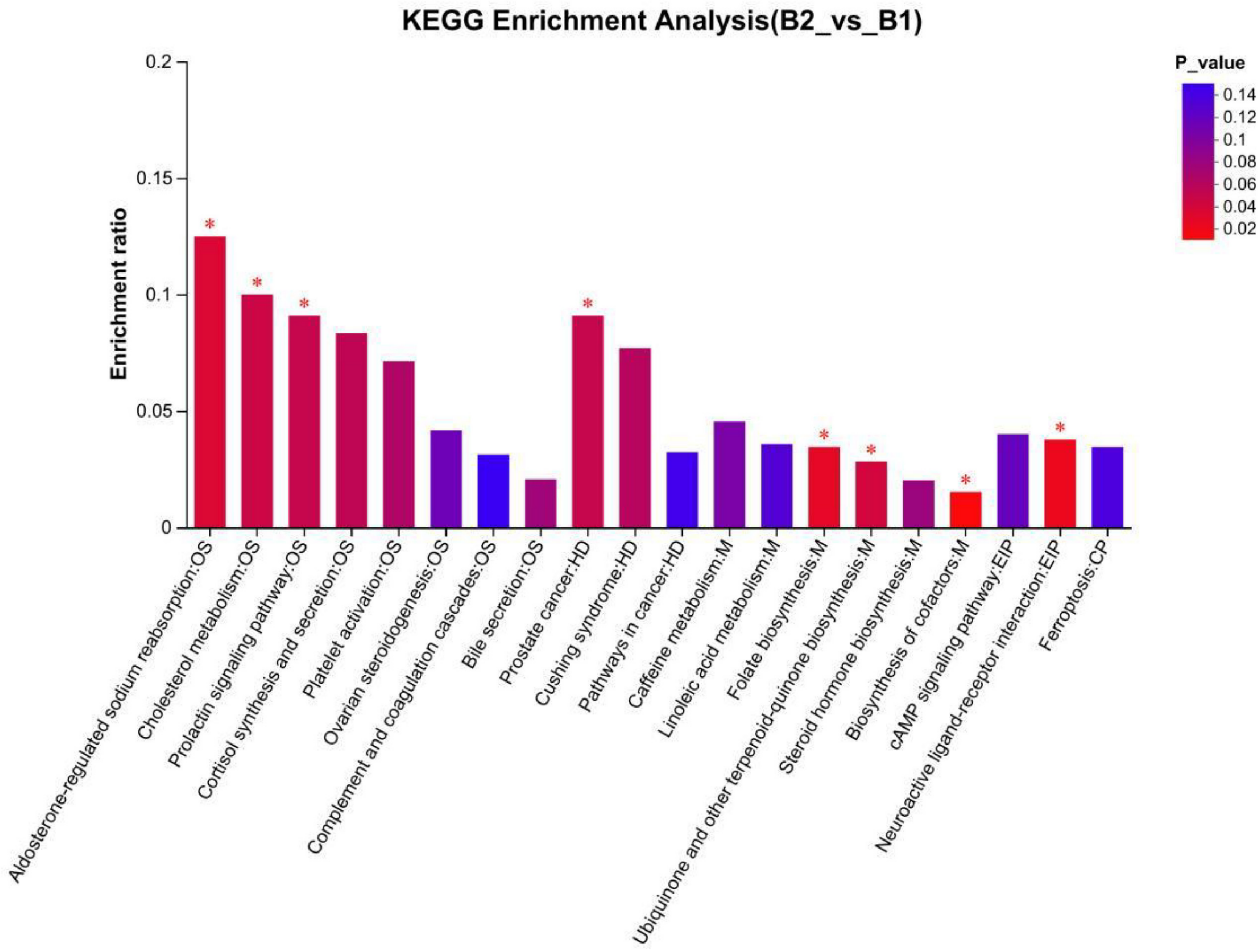
KEGG enrichment analysis plot. B1, non-drug-resistant epilepsy (NRE); B2, drug-resistant epilepsy group (DRE). *P < 0.05.

**FIGURE 7:**
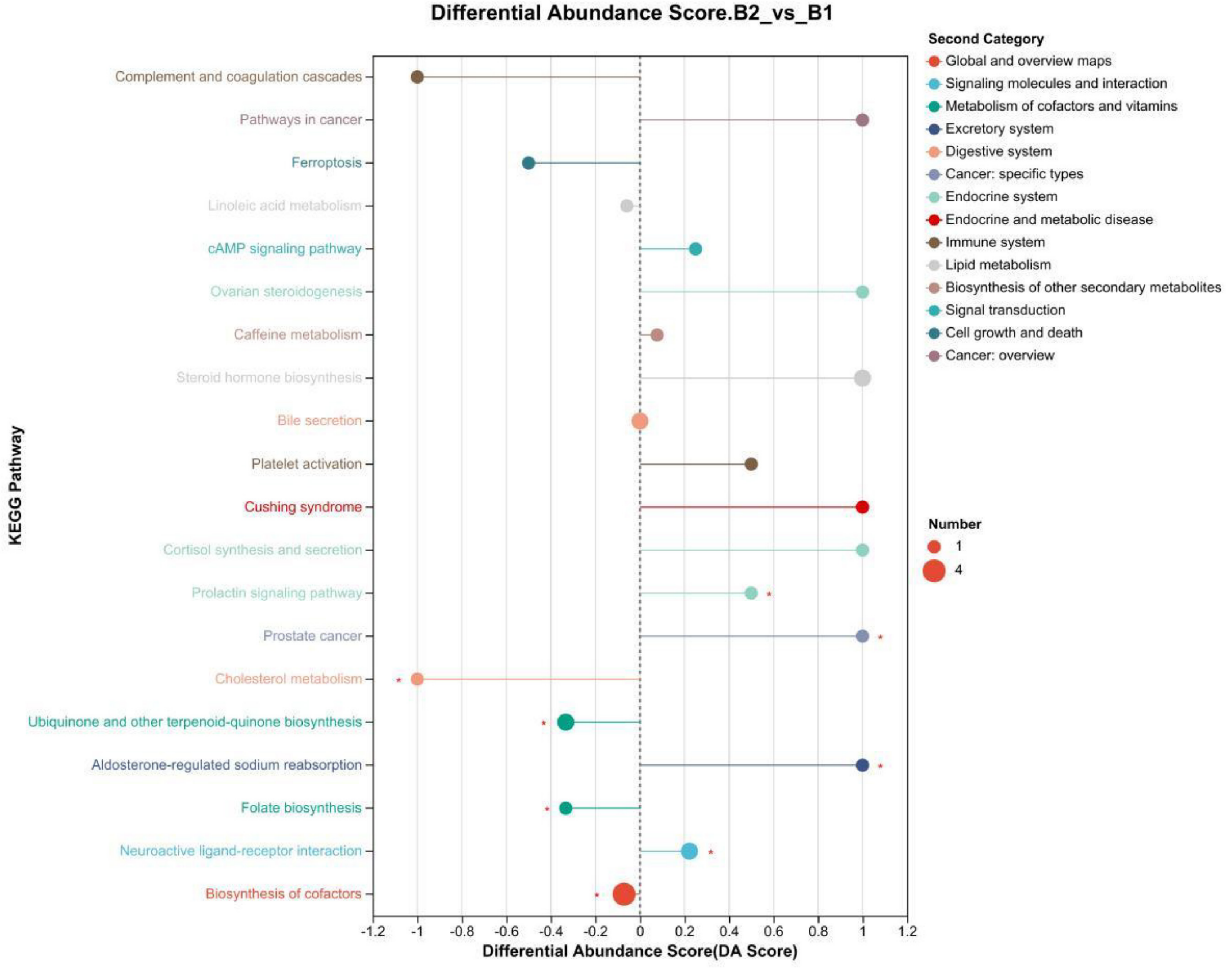
KEGG differential abundance score (DAS) plot. B1, non-drug-resistant epilepsy (NRE); B2, drug-resistant epilepsy group (DRE).

**FIGURE 8:**
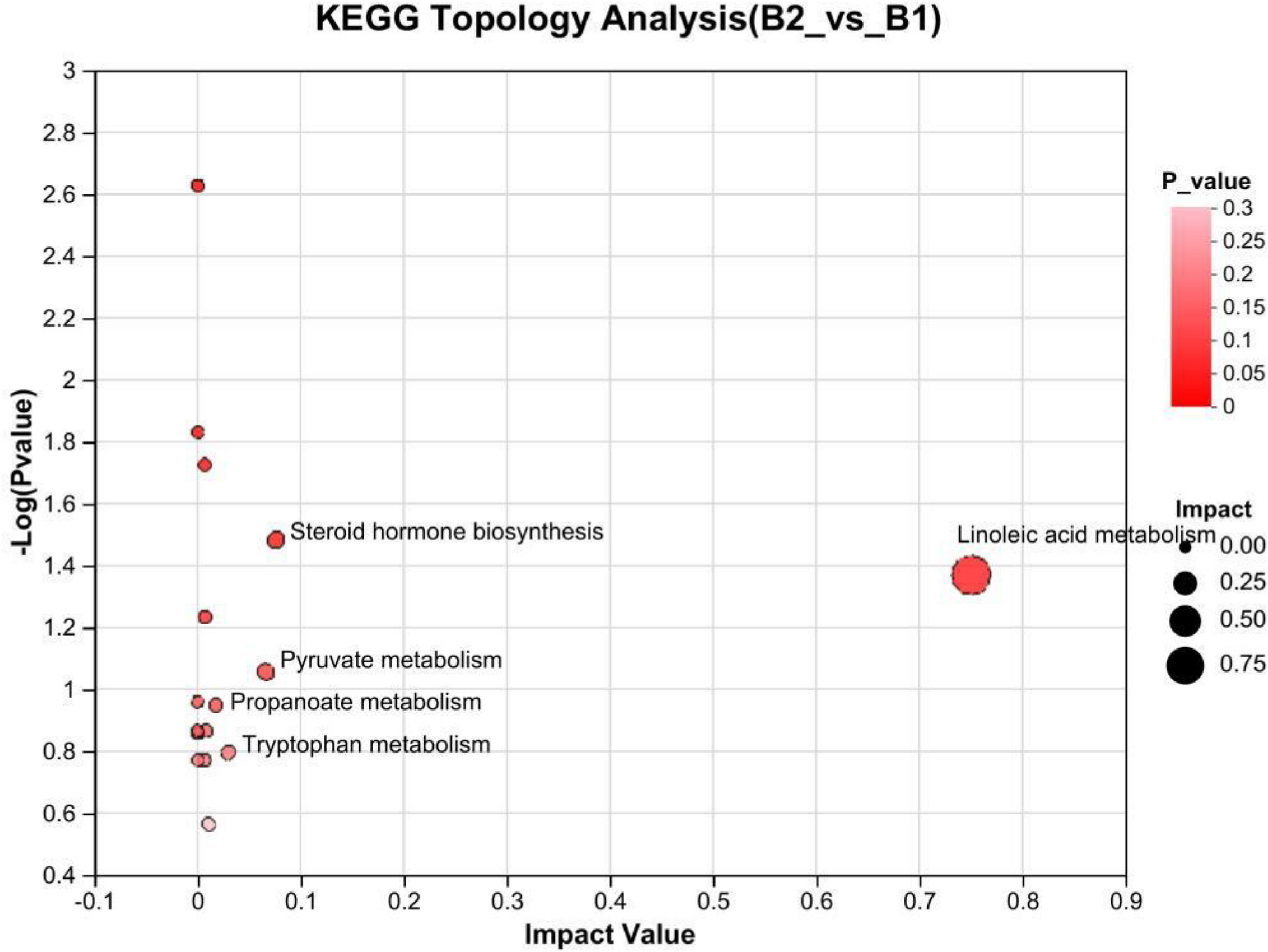
KEGG topological analysis bubble plot. B1, non-drug-resistant epilepsy (NRE); B2, drug-resistant epilepsy group (DRE).

KEGG pathways: (1) Cortisol synthesis and secretion (*p* = 0.054), differential abundance (DA) Score was 1: This pathway was significantly enriched in KEGG enrichment analysis, with the overall expression trend of metabolites in the pathway significantly upregulated (see Figures 6, 7). (2) Linoleic acid metabolism (*P* = 0.123), KEGG topology analysis pathway importance (Impact value) was 0.75: The linoleic acid metabolism pathway showed high pathway importance in KEGG topology analysis, indicating that this pathway plays a key role in lipid metabolism disorders in epilepsy patients (see Figures 6, 8). (3) Folate metabolism (*p* = 0.029): The folate biosynthesis pathway was significantly enriched in KEGG enrichment analysis, *P*-value < 0.05 (see Figure 6).

## 4 Discussion

Epilepsy is a common neurological disorder with complex etiology involving genetic, environmental, and lifestyle factors. In recent years, increasing research has focused on the role of dietary factors in the occurrence and progression of epilepsy. B vitamins, as important water-soluble vitamins, are widely involved in neural metabolism and inflammation regulation and may play a key role in the pathogenesis and development of epilepsy. This study utilized a self-established epilepsy cohort to explore the association between dietary nutritional factors (vitamins B1, B6, B9, B12) and epilepsy risk, and further analyzed the differences in these factors between DRE and NRE patients. Additionally, metabolomics was used to analyze the metabolic characteristics of epilepsy patients and explore potential underlying biological mechanisms.

### 4.1 Differences in B vitamins between NRE and DRE

To reveal differences in dietary nutrition between NRE and DRE, we conducted further research using our self-established epilepsy cohort. We found that DRE patients had lower dietary intake of vitamins B1 and B9. Both univariate and multivariate logistic regression analyses showed a negative association between dietary vitamin B9 intake and DRE risk; univariate regression analysis showed a negative association between dietary vitamin B1

intake and DRE risk, and a positive association between Dietary Inflammatory Index (DII) and DRE risk, while multivariate logistic regression analysis showed no significant correlation between vitamin B1, B6, B12 intake, DII and DRE risk; Subgroup analysis indicated that dietary vitamin B9 intake was an important protective factor for populations with low household income, higher physical activity, higher age at onset, shorter disease duration, no comorbid cognitive impairment, and no comorbid depression. Furthermore, restricted cubic spline (RCS) analysis indicated no non-linear association between dietary vitamin B9 intake and the risk of developing DRE.

Vitamin B9, also known as folate, whose active form is tetrahydrofolate (FH4), is closely related to DNA and protein metabolism and is crucial for reproductive performance in organisms. The physiological functions of vitamin B9 mainly include participating in heme synthesis, promoting fetal development, protecting cardiovascular health, and reducing the risk of dementia. The relationship between vitamin B9 and epilepsy has been studied more in pregnant women and regarding the effect of ASMs on folate concentration. Current research suggests that folate supplementation can prevent ASM-induced fetal malformations (15, 16). Patients receiving anti-epileptic drug therapy often have low blood folate levels, possibly due to ASMs interfering with folate absorption (17). The results from our self-established epilepsy cohort indicate that higher dietary vitamin B9 intake may be associated with a reduced risk of drug-resistant epilepsy in the epilepsy cohort. This finding is consistent with existing research results, suggesting a potential protective role of folate in the pathological mechanism of drug-resistant epilepsy. Folate is a key cofactor in homocysteine (HCY) metabolism. Folate deficiency leads to HCY accumulation. Studies have shown that high HCY levels lead to increased oxidative stress, exacerbating neuronal damage and potentially inducing seizures (18). Furthermore, HCY metabolites, such as homocysteine thiolactone, are neurotoxic and can modify proteins, producing N-HCY proteins that are autoimmunogenic and prothrombotic. The accumulation of such proteins may be related to epileptogenesis (19). Rasic-Markovic et al. (20) reported that folate supplementation ameliorated homocysteine thiolactone-induced seizures. Yakovlev et al. (21) conducted animal experiments suggesting that high homocysteine may increase the risk of epilepsy by disrupting the neural excitation/inhibition balance. One trial showed that folate deficiency is common in epileptic children on long-term ASMs and can lead to poor seizure control, and folate supplementation can improve epilepsy control (22). Some studies also suggest that folate supplementation in adults does not effectively reduce seizures (23, 24). Since this study involved dietary vitamin B9 and did not consider post-intake metabolic processes, interference with ASMs, etc., we randomly selected some epilepsy patients in the third part of this study for blood concentration testing of vitamin B9 and untargeted metabolomic exploration.

One notable finding is that the intake of dietary vitamin B1 also showed a negative correlation with the risk of DRE in the unadjusted model. However, after fully adjusting for comorbid depression, cognitive impairment, and quality of life (Model 3), this association was no longer significant. This indicates that the association between vitamin B1 and DRE might be mediated by its relationship with these comorbidities, rather than representing a direct, independent protective effect against drug resistance *per se*. Therefore, our results suggest that the observed insufficient intake of vitamin B1 in patients with refractory epilepsy may be more related to their higher burden of comorbidities, and these comorbidities themselves are the more direct factors driving the refractoriness of epilepsy. Future studies need to further clarify the complex causal relationship among nutrition, comorbidities, and epilepsy outcomes.

### 4.2 Metabolomic exploration

This study, through untargeted metabolomic analysis, revealed significant metabolic differences between NRE and DRE patients, further supporting the potential link between dietary nutrition and epilepsy. Given the exploratory nature of this metabolomic analysis with a limited sample size, the following findings should be interpreted as hypothesis-generating and require validation in larger cohorts.

Using the PLS-DA model, we identified 76 differential metabolites (VIP > 1.0, *P* < 0.05), some of which showed significant upregulation or downregulation in the NRE and DRE groups. These differential metabolites involve multiple metabolic pathways. Based on VIP values, *P*-values, KEGG pathway enrichment, and the focus of our preliminary research, we selected three differential metabolites—cortisol, linoleic acid, and tetrahydrofolyl-glutamate—for analysis. Their involved pathways include cortisol synthesis and secretion, linoleic acid metabolism, and folate metabolism.

Upregulation of cortisol: Cortisol, as a stress hormone, its upregulation suggests that epilepsy patients may be in a chronic stress state (25, 26). Cortisol affects nervous system excitability by activating the hypothalamic-pituitary-adrenal (HPA) axis and may exacerbate seizures (27). Previous studies have shown that chronic stress is closely related to the occurrence and progression of epilepsy. Elevated cortisol may further aggravate the pathological process of epilepsy by promoting neuroinflammation and oxidative stress (28).

Downregulation of linoleic acid: Linoleic acid is an essential polyunsaturated fatty acid of the ω-6 family (29), with anti-inflammatory and neuroprotective effects (30). Its downregulation may lead to reduced synthesis of anti-inflammatory mediators (such as prostaglandins) (31), thereby exacerbating neuroinflammation. This finding is consistent with the positive correlation of the DII, suggesting that linoleic acid deficiency may worsen the pathological process of epilepsy through the release of pro-inflammatory factors.

Downregulation of tetrahydrofolyl-glutamate: Tetrahydrofolyl-glutamate is a key intermediate in folate metabolism. Its downregulation suggests that abnormal folate metabolism may play an important role in DRE. Folate deficiency may lead to the accumulation of homocysteine (HCY), thereby inducing oxidative stress and neuronal damage (31). Previous studies have shown that HCY metabolites are neurotoxic and may increase the risk of seizures by disrupting the neural excitation/inhibition balance (19). This finding is consistent with the negative correlation between dietary vitamin B9 and DRE.

KEGG pathway enrichment analysis showed that cortisol synthesis and secretion, linoleic acid metabolism, and folate metabolism pathways were significantly enriched in epilepsy patients. Abnormalities in these pathways may affect the occurrence and development of epilepsy through various mechanisms. Elevated cortisol may exacerbate the pathological process of epilepsy by promoting neuroinflammation and oxidative stress. Future research could explore the possibility of reducing seizures by modulating HPA axis function or using cortisol receptor antagonists. Linoleic acid, as a precursor for anti-inflammatory mediators, its downregulation may worsen the pathological process of epilepsy through the release of pro-inflammatory factors. Supplementing linoleic acid or its metabolites (such as deep-sea fish oil) may become an adjunctive strategy for epilepsy treatment. Abnormal folate metabolism may lead to HCY accumulation, thereby inducing oxidative stress and neuronal damage. Folate supplementation may improve the prognosis of drug-resistant epilepsy by regulating HCY metabolism.

The negative correlation between dietary vitamin B9 intake and the risk of DRE is combined with the downregulation of tetrahydrofolate-glutamate (a key intermediate in folate metabolism) discovered in metabolomics, jointly pointing to the potential disorder of the folate metabolism pathway in DRE. We hypothesize that a lower dietary B9 intake may occur in susceptible individuals or interact with antiepileptic drugs, leading to a decrease in the efficiency of folate metabolism in the body. This may cause the accumulation of homocysteine (HCY). Elevated HCY not only has neurotoxicity itself, but also may disrupt the stability of neural networks by inducing oxidative stress and neuroinflammation. This extensive metabolic stress state may have a synergistic effect with other metabolic changes we observed in DRE patients, such as the upregulation of cortisol (a stress marker) which may exacerbate neuroinflammation; while the downregulation of linoleic acid, which has anti-inflammatory effects, may further weaken the brain’s anti-damage ability. Therefore, a lower dietary B9 intake may act as an initial factor, through perturbing the axis of folate/HCY metabolism, and together with other disrupted metabolic pathways, constitute an unfavorable metabolic environment, ultimately promoting the progression of epilepsy’s refractoriness. Of course, this hypothetical pathway requires future studies to directly measure relevant intermediate products (such as HCY) and conduct functional experiments to verify.

### 4.3 Study strengths and limitations

This study, using a self-established cohort and large-scale epidemiological data, conducted a systematic exploration of the association between B vitamins in the diet and drug-resistant epilepsy and non-drug-resistant epilepsy. The integration of non-targeted metabolomics provided new insights into the metabolic characteristics of DRE patients, suggesting a potential link between diet and neuro-metabolism. These findings regarding the possible neuroprotective effect of vitamin B9 offer new avenues for dietary intervention and provide valuable references for public health policies. However, some limitations must be acknowledged. The cross-sectional design cannot infer causal relationships (32), so future longitudinal studies are needed. The metabolomics analysis in this study was only conducted on a small sample (*n* = 12). Although we identified some interesting differential metabolites and pathways, due to the limited sample size, the statistical power of these results is relatively low, and their stability and reproducibility need to be confirmed in larger independent cohorts. Therefore, the current metabolomics findings should be regarded as preliminary and exploratory. Additionally, even though key confounding factors have been adjusted, there may still be the influence of unmeasured confounding factors. In our metabolomics analysis, we failed to collect and correct information about antiepileptic drug use, ketogenic diet, and other treatments from patients. These are key confounding factors that affect the metabolic profile and may lead to deviations in our results. Future research needs to systematically record and control these treatment variables. Finally, since our research subjects are limited to the western region of China and there are differences in diet and genetics compared to other populations, the external validity of our research results needs to be verified in other regions. Despite these limitations, this work lays a crucial foundation for future research. Future studies should focus on multi-omics integration and large-scale validation to advance the clinical translation of these findings and provide guidance for precise nutrition in epilepsy.

## 5 Conclusion

In this cross-sectional study of adults with epilepsy in western China, dietary vitamin B9 intake was negatively associated with the risk of DRE. For every 100 μg increase in dietary vitamin B9 intake, the risk of DRE decreased by 22%.

Metabolomic analysis revealed metabolic reprogramming characteristics in DRE patients. Key differential metabolites (such as linoleic acid, cortisol, tetrahydrofolyl-glutamate) and pathways (such as linoleic acid metabolism, cortisol synthesis and secretion, folate metabolism) provide new perspectives for elucidating the pathological mechanisms of DRE.

## Data Availability

The raw data supporting the conclusions of this article will be made available by the authors, without undue reservation.
